# Encapsulated Limonene: A Pleasant Lemon-Like Aroma with Promising Application in the Agri-Food Industry. A Review

**DOI:** 10.3390/molecules25112598

**Published:** 2020-06-03

**Authors:** María Dolores Ibáñez, Noelia M. Sanchez-Ballester, María Amparo Blázquez

**Affiliations:** 1 Departament de Farmacologia, Facultat de Farmàcia, Universitat de València, Avd. Vicent Andrés Estellés s/n, 46100 Burjassot, València, Spain; mijai@alumni.uv.es; 2 ICGM, University of Montpellier, CNRS, ENSCM, 34090 Montpellier, France; noelia.sanchez-ballester@umontpellier.fr

**Keywords:** limonene, agri-food industry, antimicrobial, herbicidal, antioxidant, encapsulation

## Abstract

Limonene, mainly found as a major component in *Citrus* spp., has been proven to possess a valuable potential as sustainable replacement to synthetic pesticides and food preservatives. This review intends to give a clear overview of the principal emerging applications of limonene in the agri-food industry as antimicrobial, herbicidal and antioxidant agent. To successfully use limonene in a greener agri-food industry, its preservation had become a top concern for manufacturers. In order to elucidate the most efficient and sustainable manner to encapsulate limonene, the different techniques and materials tested up to the present are also reviewed. In general, encapsulation conserves and protects limonene from outside aggressions, but also allows its controlled release as well as enhances its low water solubility, which can be critical for the discussed applications. Other parameters such as scalability, low cost and availability of equipment will need to be taken into account. Further efforts would likely be oriented to the elucidation of encapsulating sustainable systems obtained by cost-efficient elaboration processes, which can deliver effective concentrations of limonene without affecting crops and food products.

## 1. Introduction

Limonene or 4-isopropenyl-1-methylcyclohexene (C_10_H_16_) is a monocyclic monoterpene hydrocarbon naturally synthesized in many plants through the cyclisation of geranyl pyrophosphate by a monoterpene synthase [1]. It constitutes one of the most abundant monocyclic monoterpenes in the plant kingdom [2]. In fact, it has been found in more than 300 essential oils and principally in *Citrus* spp. (30–98%) [3,4].

It occurs as two optical isomers, named d- and l-limonene, as well as a racemic mixture [5,6,7]. The most common, d-limonene ((+)-limonene) is a colorless liquid with characteristic and pleasant lemon-like odor, normally obtained from the cold pressing of *Citrus* peels and pulps where it can be found at concentrations over 90% [8]. Whereas, l-limonene ((−)-limonene) is more present in other species such as *Mentha* spp. essential oils [9]. Both are common flavoring additives in cosmetics, food, industrial solvents and pharmaceuticals because of their fragrant and demonstrated harmlessness for humans [1,6,10].

Mechanical process or steam distillation techniques are typically the chosen methods to obtain limonene because they are green and non-organic solvents are involved [11]. However, other less conventional methods have also been tried in order to optimize d-limonene extraction. A high pressure-high temperature extraction (150 °C, 30 min) saved more energy, reduced extraction time and gave a higher yield of d-limonene from *Citrus* dried peels [12]. Additionally, limonene’s extraction by traditional hydro-distillation could be replaced by other eco-friendlier and time-saving alternative methods such as supercritical fluid extraction (15 MPa, 40 °C) [13].

Not only the methods, but also the extractants have evolved to greener ones. Normally, hexane has been used as the conventional solvent to obtain limonene from orange peel; however, it is considered toxic for health and the environment. Greener solvents, such as bio-based cyclopentyl methyl ether (CPME) and 2-methyl-tetrahydrofuran (2-MeTHF) have been confirmed as sustainable alternatives increasing limonene yield by 4 and 2-fold, respectively, in comparison to hexane [14]. Interestingly, the resulting limonene itself represents a green alternative to replace hazardous petroleum-based chemicals as *n*-hexane in the extraction or synthesis of other bioactive compounds [2,15,16,17,18]. Regarding this, the growing interest in natural ingredients and green chemicals drove the global limonene market to register a Compound Annual Growth Rate (CAGR) of 4.2% during the 2018–2023 forecast period [19].

Limonene’s high availability in nature and proved safety make it widely exploitable as flavoring agent and adjuvant in food and beverage industries, as well as in cosmetic’s for the formulation of perfumes and other personal hygiene products [5]. However, limonene is not only an agreeable sensation in commercial products; it has also demonstrated, alone or in combination with other substances, a broad-spectrum of health benefits, including anti-cancer, anxiolytic, anti-inflammatory, antioxidant, analgesic, antidiabetic and antiallergic activities [6,16,20,21,22]. Furthermore, other renewable applications of limonene are rapidly expanding these days, especially in the agri-food industry since the publication of its limited insecticidal activity in 1988 [23]. Between them, interesting to highlight its potential as an antimicrobial, herbicidal and antioxidant agent [24,25]. Phytochemical bio-pesticides have generally demonstrated to be environmentally friendly and safe for human and non-target microorganisms [26,27]. In particular, the risk of limonene to non-target arthropods, earthworms, soil microorganisms and terrestrial non-target plants has been considered noticeably low [28,29,30,31,32,33].

Furthermore, its limited insecticidal activity can be increased by its demonstrated synergetic effects when combined with other aromatic compounds such as linalool, camphor and isoeugenol [34]. For instance, the synergic action of *R*-(−)-limonene/(−)-borneol against the larvae *Spodeptera littoralis* caused a mortality of 84.6% when only 17.6% was expected. This synergic capacity with multiple natural substances opens divers possibilities of developing new formulations with biological efficacy in the agri-food field.

Nonetheless, the employment of limonene presents certain limitations coming from its instability, fragility and volatile nature. In fact, it can be easily degraded if it is not well protected from external factors like oxygen, light and temperature when applied. In this sense, any variation in the temperature could have a significant effect in the activity of the essential oil [35]. Particularly, the influence of the storage temperature in both stability and biocidal activity of thyme and citrus essential oils has been established [36] exhibiting post-application temperature an important influence in the insecticidal efficacy of *T. vulgaris* essential oils [37] that could be extended to other biological properties.

To obviate these limitations and be able to increase its action duration and provide a controlled release as well as improve its activity, many studies have been focusing on finding the most suitable materials to encapsulate limonene.

Therefore, in the present article we have revised the studies that collect information regarding the antimicrobial, herbicidal and antioxidant activities of limonene in the agri-food industry, with the objective to demonstrate its potential as a bio-alternative to synthetic pesticides and preservatives. Moreover, the materials and techniques tested until the moment to encapsulate limonene, as well as their characteristics and properties, have been also reviewed in order to discover the most efficient and sustainable manners to start applying limonene in a greener agri-food industry.

## 2. Results and Discussion

### 2.1. Applications of Limonene in the Agri-Food Industry

#### 2.1.1. Prevention and Inhibition of Pest Attack in Crops and Food-Spoilage Microorganisms

Currently, 137 pathogens and pests have been associated with yield losses in the basic crops worldwide—wheat, rice, maize, potato and soybean, being especially remarkable in food-deficit regions with fast-growing populations, and frequently with emerging or re-emerging pests and diseases [38]. These microorganisms have detrimental effects on the shelf-life, physical characteristics and quality of the food products; also causing serious economic losses. Therefore, the prevention and/or inhibition of microbial contamination of crops and food products are an important challenge for the global agri-food industry. In response to this, there is an increasing interest in natural antimicrobial products, which could avoid pest attack in crops, as well as food spoilage pathogens and extend storage life [39,40].

Concretely, limonene represents a safer and “greener” alternative to commercial synthesized antimicrobial products whose environmental and human health safety are disputed. In fact, Ünal et al. demonstrated the broad-spectrum and dose-dependent antifungal effect of limonene, showing higher effectiveness than standard product Fungizone^®^ at even lower doses (10 µL) [41]. Even more, limonene can modulate the antimicrobial effect of commonly used antibiotics against certain strains [42]. As an example, the combination of limonene and gentamicin considerably reduced the minimum inhibitory concentration (MIC) versus both Gram-positive and Gram-negative clinical bacteria, reaching values of 13.7–4 µg/mL against *S. aureus* and 30–20.1 µg/mL, against *E. coli,* respectively [43]. However, other standard antibiotics such as levofloxacin have shown greater antibacterial efficacy against *Listeria monocytogens* colonies [44].

It is important to highlight that the antimicrobial efficacy of limonene may vary according to its stereochemistry and the target pathogen. Proof of this was given by the difference of at least three-fold in the antibacterial effect against *Staphylococcus aureus* between enantiomeric forms of limonene. Different MIC values were observed when testing d- and l-limonene individually against the foodborne pathogens *Escherichia coli* (ATCC 11775) and *S. aureus* (ATCC 12600); and the racemic mixture exhibited lower MIC (8 mg/mL) than both enantiomers separately (27 mg/mL) versus the food contaminant *Enterococcus faecalis* [45].

Several studies also pointed temperature as an important factor in the magnitude of antibacterial activity of limonene. Particularly, bacterial membrane seemed to be more sensitive to limonene at lower temperatures. These observations may be related to the high volatility of the monoterpene hydrocarbon at increasing temperatures [46]. Nevertheless, the bactericidal activity of limonene against *E. coli* was improved with simultaneous applications of heat and acidic pH (4.0), increasing outer membrane permeability and altering β-sheet proteins [47]. The effect of limonene on bacterial cell membrane permeability has been recently corroborated for *L. monocytogens* [44]. Results showed that an optimal dose of two MIC for 6 h at 37 °C caused severe morphological modifications in the cell morphology, increased membrane conductivity and caused alterations in the respiratory and ATP synthetic chains, producing respiratory metabolic disorders and finally, death [44].

Limonene has reported as the lowest MIC (0.421 mg/mL) and minimum bactericidal concentration (MBC; 0.673–1.682 mg/mL) when compared with other hydrocarbon (α-pinene, myrcene) and oxygenated (geraniol, linalool, nerol and terpineol) monoterpenes against the Gram-positive food-spoiling bacterium, *S. aureus*, and the two Gram-negative bacteria, *E. coli* and *Salmonella enterica* [48]. However, limonene’s MIC and minimum fungicidal concentration (MFC) were lower (0.75 and 3 µL/mL) than those observed in the oxygenated compounds citral (0.188 and 0.375 µL/mL) and eugenol (0.4 and 0.8 µL/mL) against *Zygosaccharomyces rouxi*, responsible of the spoilage of apple juices and high sugar foods [49]. In general, limonene has shown stronger antifungal effect (EC_50_ 238 mg/mL) than other hydrocarbons, like 3-carane (EC_50_ 259 mg/mL), myrcene (EC_50_ 288 mg/mL) and β-cymene (EC_50_ 1051 mg/mL) against the aflatoxin-producing fungus *Aspergillus flavus*. However, this activity is lower compared to the oxygenated monoterpenes, like citral (EC_50_ 212 mg/mL), citronellol (EC_50_ 87 mg/mL) and the aromatic compound thymol (EC_50_ 20 mg/mL) [50].

The antibacterial activity of limonene can be enhanced by the additive effect of other compounds. Although limonene has been reported as one of the principal contributors of the antimicrobial activity of *Citrus* spp. essential oils [51], synergistic effect of minor compounds of finger citron (*Citrus medica* L. var. *sarcodactylis*) essential oil enhanced the bactericidal activity against common foodborne bacteria *E. coli*, *S. aureus*, *Bacillus subtilis* and *Micrococcus luteus* [52]. This additive effect was also detected against three spoilage bacteria (*Lactobacillus plantarum*, *L. brevis* and *B. coagullans*) and fungi (*Saccharomyces bayanus*, *Pichia membranifaciens* and *Rhodotorula bacarum*) of fruit juices [53]. However, limonene showed higher a antimicrobial effect compared to orange extract with mucilages and glycosides against *Candida albicans*, *A. niger*, *Aspergillus* sp. and *Penicillium* sp. [54]. Similarly, dl-limonene completely inhibited the growth of *A. niger* and the aflatoxin production at 500 and 250 ppm, respectively, concentrations at which neither *C. maxima* and *C. sinensis* essential oils nor their combination reached a total inhibition [55].

On the basis of the studies mentioned above, we can conclude that limonene possess acceptable antimicrobial activity, even higher than other terpenes, extracts and/or essential oils against a broad range of bacteria and fungi; making limonene a promising antimicrobial candidate to be employed in the current, more sustainable agri-food market.

#### 2.1.2. Herbicidal Activity

The control of weeds and other unwanted plants in a cost-effective manner is crucial to agriculture and related industries as they are responsible of the highest potential losses in productivity (34%) compared to animal pests and pathogens (18% and 16%, respectively) [56,57]. As a response, there are more than 200 herbicidal compounds commercially available worldwide, being herbicides the widest pesticide product traded accounting for 47.6% of the global pesticide sales followed by insecticide (29.4%), fungicide (17.5%) and others (5.5%) [58]. According to Statistics MRC (Market Research Companies), the Global Herbicides Market accounted for $27.45 billion in 2016 and the demand for herbicides in agriculture is still expected to increase around the world reaching $44.56 billion by 2023 [59]. The overuse of synthetic herbicides leads to a rapid spread of herbicide resistant weeds [60]. Resistant problems have been reported in 262 species (152 dicots and 110 monocots) affecting 92 crops in 70 countries [61].

Sustainable weed management based on natural products research has led to discover new herbicides as well as new modes of action [62,63]. Although only a small fraction of the world’s plant biodiversity has been screened for herbicidal activity until now, interesting herbicidal compounds with novel mechanisms of action have been discovered. Among these natural products, volatile compounds have been extensively investigated as sources of efficient and safer herbicides for human health and environment [64,65,66].

Particularly, limonene has demonstrated a broad-spectrum phytotoxic potential. It has been one of the most active monoterpenes evaluated against the seed germination and primary radicle growth of radish (*Raphanus sativus* L.) and garden cress (*Lepidium sativum* L.) achieving a significant inhibition of their germination and root elongation (10^−4^–10^−3^ M) [67]. Limonene also showed stronger herbicidal activity facing *Arabidopsis* plants than other monoterpenes like citral, carvacrol and pulegone [25]. However, a comparative study of the weedicide activity of key lime (*C. aurantiifolia* Christm.) essential oil and its main compounds limonene (40.92%) and citral (27.46%) facing three important monocot weeds: *Avena fatua* L., *Phalaris minor* Retz. and *Echinochloa crus-galli* (L.) Beauv., demonstrated the low phytotoxicity of the hydrocarbon monoterpene with respect to the whole essential oil and the oxygenated monoterpene [68], corroborating the fact that monoterpene hydrocarbons usually exhibit less potent allelopathic activity than oxygenated ones [67,68,69,70,71,72,73].

Additionally, interesting results were obtained with limonene against the cosmopolitan weed slender amaranth (*Amaranthus viridis* L.) whose germination, seedling growth, dry weight as well as chlorophyll content and cellular respiration were significantly affected by this monoterpene hydrocarbon. Limonene totally inhibited the germination of *A. viridis* at a concentration of 7 µL, and it reduced the radicle length between 70% and 90%, as well as the seedling dry weigh by about 17% and 33% at only 1 and 5 µL, respectively [74].

Foliar application of d-limonene at concentrations of 100 and 200 kg ai/ha produced the death of certain weed species, being especially sensitive to d-limonene velvetleaf (*Abutilon theophrasti* Medik.), Indian jointvetch (*Aeschynomene indica* L.), barnyard grass (*Echinochloa crus-galli* (L.) Beauv) and southern crabgrass (*Digitaria ciliaris* (Retz.) Koel) experimenting death after three days of treatment with 50 kg ai/ha of d-limonene [75].

The mechanisms by which limonene and other terpenes affect germination and/or growth of plants are still not well known. It has been observed that solubility may be a key factor implicated in phytotoxicity. Interestingly, relatively more lipophilic monoterpenes have showed less phytotoxic activity than more water-soluble ones in inhibiting seed germination and/or primary root growth, although they had higher activity on the oxidative metabolisms of mitochondria. In this sense, limonene did not affect the primary root growth of maize (*Zea mays* L.) at none of the concentrations (0.1–10.0 mM) assayed, whereas more hydrophilic oxygenated monoterpenes like camphor and eucalyptol affected the synthetic capacity of root cells and consequently, showed considerable potency in reducing both fresh and dry weights of primary roots. On the other hand, limonene caused more detrimental effects on mitochondrial respiration at increasing doses than more hydrophilic monoterpenes, even reaching abolition of respiratory control between 1.0 and 5.0 mM. This could be a consequence of the higher lipophilicity of limonene, which allows a better penetration through mitochondrial membranes [76].

On the other hand, an increased lipid peroxidation was observed with limonene on the root growth of maize, causing high inhibition values of 73.69–90.10% in the radicle elongation from 24 to 96 h of treatment [70]. So, lipid peroxidation may also be a possible mechanism of action by which limonene and essential oils that contain it can exert its phytotoxic activity [55]. Finally, limonene also showed a strong antimicrotubule activity at high and low dosages, provoking the breakage and leakage of the plasma membrane and finally causing plant death [25].

It is interesting to note that the different herbicidal activity of a compound against a selected weed may be due to a different mechanism of action. As illustration, limonene influenced the photochemical processes in carrot cultivar Splendid deriving in a lower shoot and root biomass, while it reduced gas exchange in cultivar Parano resulting in lower stomatal conductance. While for cabbage, cultivar Lennox showed better tolerance and fast recovery to limonene than cultivar Rinda by means of developing photochemical processes of increasing efficiency that provide energy for defense and repair action [77].

The photochemical processes of limonene, have been also studied in the algae *Chlorella vulgaris* (Chlorophyceae), showing that this monoterpene hydrocarbon caused a drastic degradation of the photosynthetic pigments, among them xanthophyll at 1.6 mM [78].

Unfortunately, limonene such as other monoterpenes and essential oils exerts a non-selective phytotoxicity, affecting not only weeds, but also cultivated plants [79,80]. Both leaves of cabbage (*Brassica oleracea* L.) and carrot (*Daucus carota* L.) were directly damaged at limonene’s concentrations higher than 90 and 120 mL/L [77]. Thus, it may be necessary to evaluate previously the threshold concentration of limonene for cultivated plants with the aim to avoid any harm for them. Consequently, limonene has been included as the main active principle in several non-selective herbicidal formulations [81,82].

#### 2.1.3. Antioxidant Activity

Stored food products are subjected to free radical generation consequence of oxidative stress. Natural preservatives to combat this deterioration represent eco-alternatives to synthetic phenolic antioxidants such as *tert*-butylhydroquinone (TBHQ), butylated hydroxyanisole (BHA), butylated hydroxytoluene (BHT), and propyl gallate, safer for human health and the environment [83]. Limonene is thought to be a possible substitute to these commonly used synthetic antioxidants, particularly in increasing the oxidative stability of vegetable oils in the deep-frying process without affecting the sensory properties of the fried products [84]. As previously mentioned, in other cases, the antioxidant potential of limonene is still significantly lower than other reference antioxidants, such as trolox, concretely at the range of concentrations between 2 and 2000 μM [85].

The natural presence of limonene in certain foods can represent a quality indicator. For instance, the loss of limonene in *Citrus* during their storage would affect the original flavor and aroma of the product and consequently, a deterioration of the food [86]. In other food products, the ability of limonene to repress the formation of reactive oxygen species (ROS) such as hydrogen peroxide (H_2_O_2_) and glutathione (GSH) has been confirmed [85,87]. These results demonstrate the disposition of limonene to avoid food degeneration, indicating its usefulness in overcoming storage losses and enhancing the shelf-life of food products. Moreover, the 2,2-diphenyl-1-picrylhydrazyl (DPPH) free radical scavenging effect and reducing power of limonene have been confirmed to be even higher than the antioxidant activity exerted by other terpenes like nerol, terpineol, geraniol, linalool and myrcene [48]. This activity can even be enhanced to an IC_50_ of 116 ppm when encapsulating the monoterpene hydrocarbon in chitosan-NaTPP, as this material protects limonene from degradation and increases the solubility in water [88].

*Citrus* spp. with remarkable concentrations of limonene enjoys great antioxidant activity, representing potential natural, eco-friendly and safer alternatives to synthetic preservatives in food packaging and preservation [89,90]. In this way, *C. sinensis* (90.66% limonene) and *C. limon* var. *pompia* (*pompia*; 256.3 mg/mL limonene) have demonstrated a good ability in scavenging radicals [55,91]. The antioxidant activity of *C. aurantifolia*, *C. limon* and *C. paradisi* (40.16%, 57.20% and 73.5% of limonene, respectively) essential oils occurs in a limonene dose dependent manner, reaching *C. paradise* essential oil values of 84.92% ± 0.5% and 92.45% ± 0.6% in the DPPH and β-carotene-linoleic acid assays, respectively [92].

Limonene’s termination-enhancing antioxidant chemistry, shared with other compounds like linalool and citral, might be relevant in food preservation. In this case, the concentration of the terpene compound as well as the characteristics of the substrate would be limiting factors in its antioxidant activity [93].

All in all, limonene has well proven its potential to be used as innocuous and sustainable preservative in food processing, storage and packaging. Nevertheless, the encapsulation of this natural alternative to synthetic preservatives is a fundamental step to keep its activity and avoid its oxidation.

### 2.2. Limonene Encapsulation Techniques

**Figure 1:**
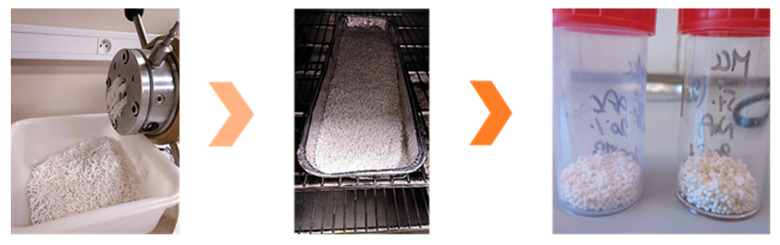
Extrusion-spheronization techniques with pine essential oil (α-pinene 26% and limonene 19%) [95].

Several encapsulation processes have been developed and reported in the literature in order to encapsulate and protect fragile compounds such as limonene. The efficacy of the encapsulation towards Limonene’s oxidation, evaporation or controlled release would generally depend on the chosen encapsulation methodology (atomization, extrusion, fluidized bed, coacervation, etc.) and on the wall materials used. For instance, retention of non-encapsulated d-limonene in extruded starch was much lower (8.0%) than for d-limonene encapsulated with β-cyclodextrin (92.2%) and sodium caseinate (67.5%) capsules [94]. In the mixtures without encapsulation, the protection seems to be provided by the interactions formed between starch and d-limonene through inclusion complexation. These interactions can be increased by changing the wall material used in the extrusion process and reducing the working temperatures. Thus, a pine essential oil’s retention of 63.19% with limonene and α-pinene as the main compounds was achieved when starch was replaced by microcrystalline cellulose and less severe temperatures conditions were employed (process performed at room temperature (r.t.)) during extrusion (Figure 1) [95].

Hence, in the following part of this review, representative techniques of limonene encapsulation: simple and complex coacervation; nano- or microencapsulation using different wall materials such as polysaccharides, proteins or inorganic carriers; molecular inclusion with cyclodextrins; spray drying; electrospinning and nanoemulsions were discussed. Other alternative methods such as electrospraying and supercritical fluid technology, less commonly used for the encapsulation of limonene, were also included.

#### 2.2.1. Coacervation

Coacervation is one of the oldest and most widely used techniques of encapsulation, which can be divided into simple and complex coacervation. While the first one implies the use of one colloidal solute such as chitosan [96]; in complex coacervation encapsulation occurs from the interaction of two oppositely charged colloids, such as pectin-whey proteins or gelatin (gum Arabic or chitosan) [97,98,99]. Despite both methods have shown advantages for the encapsulation and stabilization of EOs; complex coacervation seems to be more recurrent in the literature for the encapsulation of limonene [100]. Simple coacervation has been used by Souza et al. for the preparation of insect repellent limonene oil microcapsules with chitosan [96]. One important advantage of this method is the control of the shape, size and release rate of the encapsulated limonene only varying the concentration and ratio of the chitosan and NaOH solution.

The most common composite matrices employed as delivery vehicles for limonene in complex coacervation are made of sustainable polymers such as chitosan, pectin, gelatin, cellulose and gum acacia or Arabic [97,98]. These composite matrices have shown higher limonene encapsulating efficiency (EE) than chitosan crosslinked with sodium tripolyphosphate (46% vs. 51.3%; 89.7% and 98.6% for chitosan-cellulose and gelatin-gum Arabic, respectively) [88]. Release profiles are typically done in two phases, an initial phase of 24 h characterized by a burst release effect, probably due to the release of limonene found on the surface of particles, followed by a decrease release, which can go over 162 h.

One of the potential problems of complex coacervation is the weak mechanical resistance of the coacervates, due to polymer’s water solubility, making them inappropriate for applications where a long shelf-life and a good mechanical strength are required. Although crosslinking agents such as formaldehyde or glutaraldehyde can be used to enhance the stability. They possess drawbacks due to their toxicity and potential side reactions between the encapsulated material and the residues of the crosslinking agents. A safer alternative to aldehyde-based crosslinking agents, is to use tannins or sodium tripolyphosphate as crosslinkers [101].

Complex coacervation, using tannic acid as a hardening agent, was employed for the preparation of chitosan/gum Arabic microcapsules of limonene [101]. Mono or polynuclear structures were obtained depending on the emulsifier used (Span 85 or polyglycerol polyricinoleate (PGPR)). The obtained microcapsules shown sustained release pattern, with a cumulative release of limonene after 7 days at 37 °C ± 1 °C of 75% and 52% for the polynuclear and mononuclear microcapsules, respectively. The best EE% (98.6%) was achieved with Span 85. These values are in agreement with those obtained by Rabisková et al. who stated the preference of hydrophobic substances for emulsifiers with low hydrophilic-lipophilic balance (HLB) values (Span 85—HLB of 1.8 and PGRP—HLB of 2–4) [102].

Drying methods have also been reported to have an effect on the retention of volatile limonene and in the structure of the wall matrix influencing consequently the storage stability [103]. For instance, significantly lower retention of limonene was observed for freeze dried whey/corn fiber-limonene samples after storage caused by the diffusion of limonene through the wall materials, which are generally more porous and loose than the structures obtained by spray drying [99,104].

Encapsulation by coacervation as described in these works appears to be an effective technique for encapsulating limonene aroma providing a good barrier against oxidation of sensitive materials. The most important parameters in the preparation of coacervated biopolymers complexes with maximum encapsulation of limonene are the viscosity and the pH in order to obtain the best attractions at the highest electrostatic rate between both polymers or the polymer-protein.

#### 2.2.2. Nano(Micro)encapsulation Using Different Wall Materials

The agro-food industry has focused on developing and evaluating state-of-the-art wall materials for EOs encapsulation considering their functionality as encapsulating agents, cost, authorized grade and accessibility. Biopolymers such as polyurea, poly(vinyl alcohol) or poly(lactic acid) [105,106,107,108]; along with polysaccharides and inorganic carriers have been spotted as efficient materials for encapsulation of limonene (Table 1).

In general, the release of the limonene is mainly controlled by the initial EOs loading and the ability of the oil molecules to diffuse through the wall barrier into the surrounding environment. Interactions between the limonene molecules and the wall materials, together with the vapor pressure of the volatile substance on each side of the matrix, are the major driving forces influencing diffusion [118,119,120].

#### 2.2.3. Cyclodextrins

The α- β- and γ-cyclodextrins (CDs) have been widely studied for the encapsulation of volatile and thermo-sensible substances such as EOs essential oils for increasing their solubility, permeability and chemical stability to prevent oxidative degradation, which can reduce its utilization [121]. CDs are biocompatible, biodegradable, have the GRAS status and have been approved as additives in the European Union [122]. In general, due to its lower cost and ability to interact with a wide variety of EOs, β-CD is widely used for the encapsulation of limonene. Although the most popular method to form limonene-CDs inclusion complexes is in solution, the alternative kneading method has also been reported [123].

The determination of the stability constant of limonene inclusion complexes with CDs is of critical importance to take advantage of the complexation potential of CDs in the agro-food industry. The limonene-CDs complexation process has been modeled by 1D and 2D ROESY NMR experiments and found to be driven by non-covalent interactions. It was observed that only partial complexation was obtained, with non-complete formation of 1:1 inclusion complexes. Limonene-β-CD complex seems to be slightly more stable than limonene-α-CD with binding energies of −4.54 kcal and −4.05 Kcal, respectively [124]. A similar trend was observed by Astray et al. who determined the binding constant of limonene-(α/β)-CDs complex formation by UV-Vis technique coupled with molecular mechanics’ calculations [125].

Development of composite films based on biodegradable polymers and β-CD/limonene inclusion complexes for potential production of bio-active and biodegradable food packaging materials have gained increased interest. Incorporation of active EOs into polymers is a technological challenge, due to the need to avoid evaporation during melt processing of the polymer. Such a challenge can be solved by incorporation of the volatile limonene within CDs [126]. It is important to note that although β-CD can increase solubility, permeability and adherence of limonene to the bacterial walls, its complexation with d-limonene can result in structural changes, which can prevent the physicochemical interaction with the cellular bacterial system and therefore the complex would be no active [43].

Furthermore, β-CDs show some drawbacks that limit its application such as low aqueous solubility (1.8% *w*/*v*, at 25 °C). To improve water solubility β-CD derivatives like 2-hydroxylpropyl-β-CD (HP-β-CD; 50% *w*/*v*, at 25 °C) have been synthesized [127]. Encapsulation efficiency was found to be monoterpene chemical nature dependent. While oxygenated terpenes such as eucalyptol and thymol were entrapped with an EE > 82%, monoterpene hydrocarbons like limonene presented lower EE (from 15% to 25%). The lower EE% values obtained for monoterpene hydrocarbons have been associated to their very low water solubility.

Molecular inclusion of limonene into CDs can be found in the literature combined with other techniques such as extrusion, electrospinning and spray drying. The association of techniques can yield materials presenting superior mechanical properties in which the inclusion complexes formed with CDs permit the efficient preservation of limonene [128,129,130].

#### 2.2.4. Spray Drying

Spray drying is a well-known technique widely used in agro-food and pharmaceutical fields due to its low cost and availability of the equipment. It is a physical encapsulation technique used in the protection and release of unstable active materials confined into polymeric matrices. It is important to highlight that the spray drying operating conditions and powder properties are going to be critical for obtaining encapsulated compounds displaying controlled release function and stability during the storage [131,132].

While gum acacia, gum Arabic and modified starches have been the most commonly used wall materials for spray drying encapsulation methods in the past [133,134,135]. Currently, other wall materials like maltodextrin or whey and soy protein are investigated as alternative sources [136,137]. It is interesting to remark that although traditional materials seem to still give the highest flavor retention, soy and whey protein materials have demonstrated to effectively limit the oxidation of limonene [136].

One of the drawbacks of using spray drying for the production of dry flavorings is the high temperatures used during processing, which can lead to the loss of volatile molecules. An interesting solution to reduce volatilization of d-limonene during spray drying process is to use a multilayer emulsion multilayer as the encapsulation system [138]. Stable emulsions were developed by combining proteins from a lupin crop AluProt-CGNA coated with chitosan and sodium alginate or pectin. Emulsions stabilized with two and three-layers of polysaccharides presented greater retention of d-limonene and physico-thermal stability (30–90 °C) than mono-layered membranes. This point is crucial for food emulsions that undergo some form of thermal processing during their production, storage or utilization such as pasteurization, sterilization or cooking. Moreover, these emulsions showed the higher aroma retention after 45 days’ storage.

CDs have also been used in combination of spray drying for flavor and aroma encapsulation [139]. While the use of a coating material for the preparation of CD/limonene spray dried powders improved the powder properties at expenses of decreasing the limonene content [140]. Dried forms of different CD/Limonene (α-, β- and γ- and HP-β-CDs) were prepared by spray drying and studied for increasing the flavor and shelf-life of non-alcoholic beverages [141]. Among the CDs tested, β-CD was the more suitable for limonene complexation and retention (66% of encapsulation efficiency and 6.25 *w*/*w* of limonene load). Furthermore, accelerated aging analysis showed that limonene content decreased less in the presence of β-CD with 40% of the complexed limonene remaining in the beverage after 9 simulated months of storage.

#### 2.2.5. Electrospinning

The introduction of highly volatile EOs within polymeric nano-scaled fibers obtained by electrospinning is a favorable route for efficient and simple encapsulation of temperature-sensitive materials. The possibility of obtaining a fibrous mesh containing the EO of choice allows the conformal and homogeneous deposition of the EO and limits the amount of material to be produced. This is not always possible with EOs-containing microcapsules, which tend to agglomerate. Electrospinning is an up-scalable process thus numerous applications can be conceived such as food packaging and fragrance release [142].

The influence of the electrospinning process parameters on the encapsulation of (*R*)-(+)-limonene with poly(vinyl alcohol) (PVA) has been studied by Camerlo et al. [143]. It was found that while temperature increases the evaporation rate of limonene, humidity affects the permeability of the polymer fibers. Polymer concentration can also influence the EE of limonene. Greater EEs were obtained from 9% PVA/limonene emulsions compared to the other PVA lower contents [144]. Higher polymer concentrations caused either an increase in the viscosity of the emulsion or the polymer precipitation, decreasing or even preventing the possibility for the encapsulated limonene to diffuse.

A combination of electrospinning and CDs complexation techniques has also been reported. The association of these two techniques allows the production of very thin fibers with large surface areas and superior mechanical properties, which permit the efficient preservation of the EOs. Fuenmayor et al. used these two techniques for the encapsulation of *R*-(+)-limonene in edible nanofibers obtained from pullulan and β-CD emulsions [130]. The critical role that plays the relative humidity on the limonene release was also highlighted by the authors. It was reported that release was taking place at values of water activity higher than 0.9. These results make this system interesting for active packaging applications, in particular for fresh foods, for which the risk of microbial degradation increases at high water activity conditions.

Interesting also to highlight the application of l-limonene as a green and non-toxic solvent alternative for the production of polystyrene (PS) fiber matrix by electrospinning [145]. Electrospinning PS is limited to organic toxic solvents, which cause environmental problems and limit its use in food-based applications. Following this strategy, controlled delivery systems for sustained release of bovine serum albumin for food-related applications were fabricated via emulsion electrospinning. Fibers showed a prolonged release period of 50 days controlled by the molecular weight of PS polymer.

#### 2.2.6. Nanoemulsions

Nanoemulsions have proven high potential application in the encapsulation of food ingredients and in the enhancement of the antimicrobial activity of EOs. Both, low- and high-energy methods have been reported for the encapsulation of limonene (Table 2).

#### 2.2.7. (Nano)-Emulsion Stabilizers

Emulsifiers have been used in the agro-food industry to create metastable emulsion-based products, which stabilize highly reactive, volatile and/or hydrophobic substances. The food industry had vastly used synthetic emulsifiers, such as sorbitan or sucrose esters and fatty alcohol ethoxylates due to their efficiency. However, synthetic surfactants can increase the incidence of human health diseases such as allergies and also environmental issues. To solve this, sustainable emulsifying agents from plants such as mucilage from different seeds, Angum gum, sodium alginate or β-lactoglobulin have started to be used for the encapsulation of food oils and flavors such as limonene [150,151,152]. Despite the vast majority of work reported focuses on the use of conventionally based systems (surfactants and polymers), particle-stabilized emulsions also referred to as Pickering emulsions have also been used for Limonene’s stabilization. These type of emulsions are stabilized by an accumulation of dispersed particles (i.e., silica or cellulose nanocrystals) at the oil–water interface forming a mechanical barrier that protects emulsion droplets against coalescence and yields high EE% (79–100%) [153,154,155].

#### 2.2.8. Alternative Encapsulating Methods

Nanocapsules of d-limonene were obtained from electrospraying an emulsion of *Alyssum homolocarpum* seed gum (AHSG) with 0.1% of Tween 20 [142,156]. Due to the less severe experimental conditions used during electrospraying, the EE achieved was greater than those reported for d-limonene encapsulation using other methods such as spray drying (environment 50% to 90%) [135,157].

An interesting approach for d-limonene encapsulation is by using recyclable porous materials (RPMs), which are highly porous and thermally stable 3D framework structures composed of 1D hydrophobic channels [158]. Two types of RPMs materials were compared in performance to encapsulate d-limonene with modified starch (Starch-CAP@DL). Both RPMs were able to absorb a large quantity of d-limonene (200 and 150 mg/g). RPMs demonstrated prolonged release (1.5 h) of d-limonene compared to Starch-CAP@DL (80% of d-limonene released instantly). RMPs biocompatibility still needs to be improved so further work to modify RPMs with suitable ligands needs to be performed.

Finally, a quite innovative manner to impregnate or encapsulate limonene in modified starches is using supercritical CO_2_ via particle from gas saturated solutions or suspensions (PGSS) [159]. One of the best advantages of using supercritical fluid technology for encapsulating limonene is the use of relatively low temperatures, which enable the encapsulation of sensitive materials. The encapsulation efficiency of limonene was of 86% compared to conventional spray drying, which showed an efficiency of 91% for the same volatile.

## 3. Conclusions

Due to its safety, wide-range of health-promoting effects, attractive flavor and fragrance, limonene is a commonly used ingredient in cosmetic, food and beverage industries. Current studies have proved that limonene also represents a suitable ingredient for the agri-food industry. In particular, it has been demonstrated its strong antimicrobial activity against a broad-spectrum of pests affecting crops and food-spoilage microorganisms, as well as antioxidant potential to avoid post-harvest decay along processing, storage/packaging processes and extending the shelf-life of food products. In addition, limonene has also shown significant phytotoxicity facing different weeds that represent an alarming hazard for agricultural production and ecology due to their rapid growth, high competitiveness and resistance development.

Its wide range of biological activities, together with its lack of toxicity and diverse mechanisms of action, make limonene a very interesting natural bio-alternative to synthetic pesticides and preservatives for a more sustainable emerging agri-food industry. Yet, in order to develop effective products, further studies are still needed to determine the active application threshold, which would not cause a damage of the crops. Additionally, more thorough knowledge on Limonene’s action mechanisms would need to be elucidated to improve its possibilities to be applied as bio-pesticide and preservative.

Post-application conditions (temperature, light, oxygen availability, etc.) have been proven to possess an unfavorable influence on the effectiveness of limonene. So, in order to preserve its activity and improve its valorization in weed control and food preservation, different encapsulation techniques and safe-biodegradable wall materials have been tested to protect and control the release of limonene. Although, most of the different encapsulation techniques described in this review have proven to be successful in delaying Limonene’s volatility, enhancing its beneficial properties and controlling its release; further cost-efficiency analysis of the processes would be demanded to encapsulate limonene at the industrial scale.

Further perspectives in the encapsulation of limonene could include its combination with other EOs to enhance its activity by the additive effect. To achieve this, additional tests on the role of the combination substance ratios, synergic/antagonic effects, optimal concentrations and potential interactions with materials used in the encapsulation need to be still realized.

## Figures and Tables

**Table 1:** Examples of wall materials used for Limonene’s encapsulation.

Wall Material	Highlighted Results	Ref.
**Polymer**		
Polymer-blend in a HPMC:PV(OH):EC ^1^	HPMC:PV(OH):EC *w*/*w*/*w* ratio of 1:1:6. Low Limonene’s EE% due to unsaturated hydrocarbon functionality.	[109]
Acrylic adhesive polymer or natural rubber	Application as pesticide (*Solanum melongena*). Penetration rate of the active agent, imidacloprid, was enhanced 2.4 times in the presence of d-limonene. Bursting release avoided.	[110]
**Polysaccharide**		
Amylose	Amylose-limonene showed less than 5% limonene released at pH acid. At pH 6–7 burst release followed by a controlled and retarded release (6 h with 34–79% release depending on the % of amylose used in the formulation).	[111]
Chitosan	Release tested in five different food simulating liquids (aqueous solutions with 0%, 10%, 50% and 95% of ethanol and isooctane). Kinetic constants augmented with the addition of ethanol, due to the increase of Limonene’s solubility.	[112]
Functionalized chitosan	Increasing the shelf-life of strawberries during storage. Chitosan functionalized with palmitoyl chloride provided better preservation after 14 days at 4 °C. Chitosan modification increased its hydrophobicity, ensuring limonene controlled release and improved its stability and adhesion to the fruit.	[113]
**Inorganic carriers**		
Silica	Limonene oxidation and retention depended on the type of silica (chemical purity, small pore volume/diameter and hydroxylated surface area).	[114,115]
Hybrid CaCO_3_ with lecithin, sodium stearate (NaSt) and acacia gum (AG)	Particles with lecithin and NaSt presenting more hydrophobic surface retained more limonene. CaCO_3_-lecithin presented minimal loss after 3 months’ storage at r.t ^2^ Hydrophobicity was more efficient than specific surface area in increasing Limonene’s retention and absorption capacity.	[116]
**Protein**		
Corn’s Zein	Optimal limonene/zein ratio was 2.0 yielding particles with D_4.3_ of 10 µm and shell thickness of 25 nm. Maximum burst release at 30 min, followed by sustained release of environ 80%.	[117]

^1^—ethylcellulose (EC), hydroxypropyl methylcellulose (HPMC) and poly(vinyl alcohol) (PV(OH). ^2^—r.t. room temperature.

**Table 2:** Examples of Limonene’s encapsulation using low- and high-energy emulsifying methods.

Emulsification Method	Highlighted Results	Ref.
**High-Energy**		
High pressure homogenizer	d-limonene/monosterin organogel (4% *w*/*w*) presented better antimicrobial activity than free d-limonene due to the higher solubility of encapsulated limonene. Small size nanoemulsion (36 nm) droplets can easily fuse with bacterial cells.	[146]
Sonication	Nanoliposomes of d-limonene/soy or rapeseed lecithins (150 nm) were added to starch-sodium caseinate (50:50) film forming dispersions. Encapsulation prevented limonene evaporation. Antimicrobial activity against *L. monocytogens* was inhibited.	[147]
Microfluidization vs. Ultrasound	Microfluidization produced droplets of 700–800 nm with the highest retention (86.2%) of d-limonene and minimum amounts of non-encapsulated oil at the surface of particles.	[104]
**Low-Energy**		
CPI	Water/Tween 80/d-limonene system. Nanoemulsions stored at 28 °C were more stable than those stored at 4 °C.	[148]
CPI	d-limonene/nisin system showing synergistic effects against food-related microorganisms: *S. aureus, B. subtilis, E. coli* and *Saccharomyces cerevisiae*.	[149]
